# Supplementary material for: Mechanical Sensing Element PDLIM5 Promotes Osteogenesis of Human Fibroblasts by Affecting the Activity of Microfilaments
Source: Biomolecules. 2021 May 19;11(5):759. doi: 10.3390/biom11050759 (PMC8161207; doi:10.3390/biom11050759)
Supplement: Supplementary file 1 [file biomolecules-11-00759-s001.zip › Figure supplement/Illustration of supplementary data.pdf]

Illustration of supplementary data：

Supplementary data figure 1 (Figure S1): Determination of Lentiviral pre-Transduction Effect. A: Fluorescence result of empty plasmid control group with MOI =1,10,100; B: Fluorescence result of transfection experimental group with MOI=1,10,100; C: Fluorescence result when MOI = 40 and MOI = 80. C group: Blank control group; M group: Normal medium group; A group: Basic medium + HiTransG A group; P group: Basic medium + HiTransG P group; Con: Blank control group, shScr: empty plasmid Control group, shPDLIM5 :experimental group of knock-down PDLIM5; MOI: Multiplicity of infection; HiTransG A/P: Viral infection booster (Scale bar = 100 μm).

Supplementary data figure 2 (Figure S2): Structure of PDLIM5 virus vector: Ubi-MCS-3FLAG-CBh-gcGFP-IRES-puromycin.

Supplementary data figure 3 (Figure S3): Alizarin red staining of hASCs and HSFs on 21 days under osteogenic induction medium, (Scale bar = 100 μm).
