# Supplementary figures and images for: Mechanical Sensing Element PDLIM5 Promotes Osteogenesis of Human Fibroblasts by Affecting the Activity of Microfilaments
Source: Biomolecules. 2021 May 19;11(5):759. doi: 10.3390/biom11050759 (PMC8161207; doi:10.3390/biom11050759)

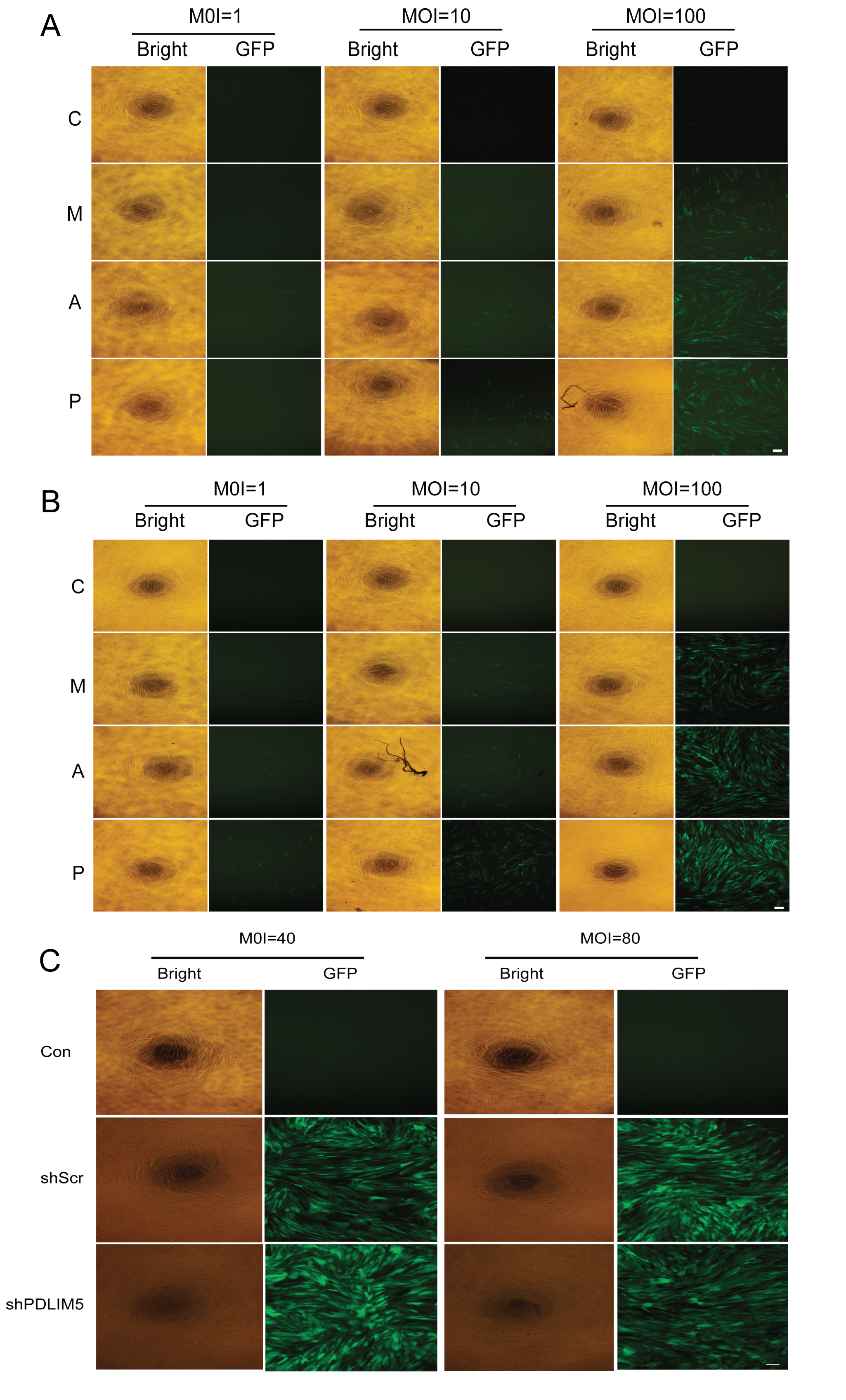

Supplement: Supplementary file 1 [file biomolecules-11-00759-s001.zip › Figure supplement/Figure S1.tif]

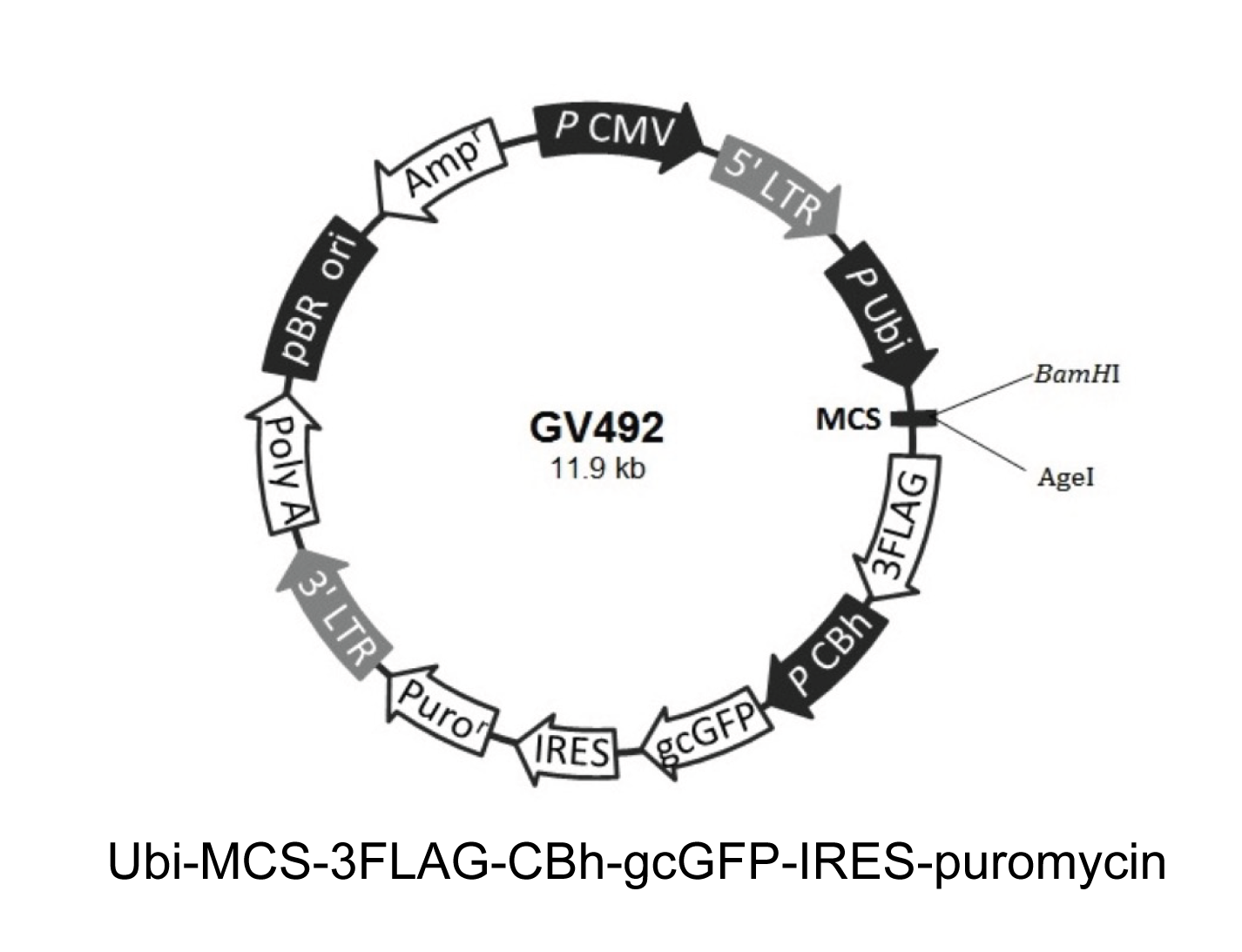

Supplement: Supplementary file 1 [file biomolecules-11-00759-s001.zip › Figure supplement/Figure S2.tif]

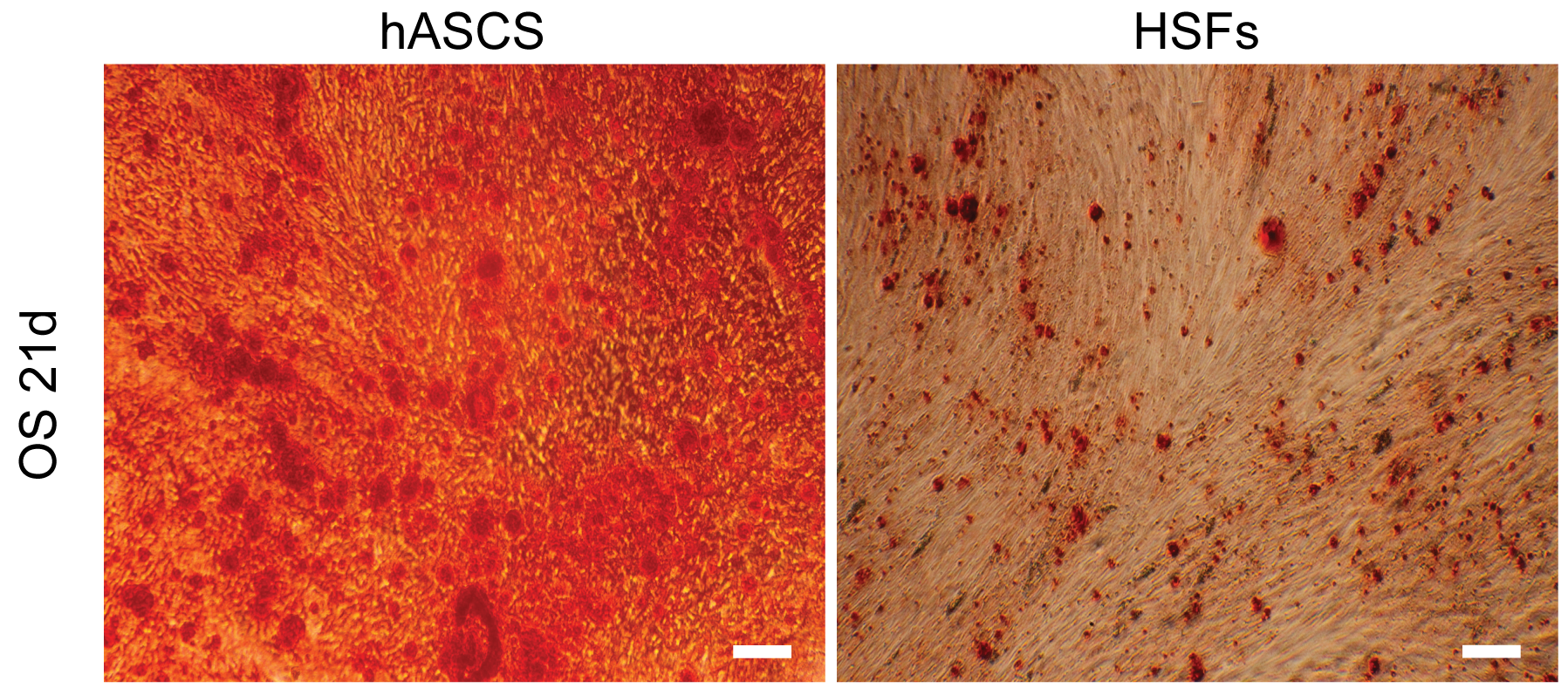

Supplement: Supplementary file 1 [file biomolecules-11-00759-s001.zip › Figure supplement/Figure S3.tif]
